# Methane formation in tropical reservoirs predicted from sediment age and nitrogen

**DOI:** 10.1038/s41598-019-47346-7

**Published:** 2019-07-29

**Authors:** Anastasija Isidorova, Charlotte Grasset, Raquel Mendonça, Sebastian Sobek

**Affiliations:** 1 Limnology, Department of Ecology and Genetics, Uppsala University, Uppsala, Sweden; 2 Laboratory of Aquatic Ecology, Department of Biology, Federal University of Juiz de Fora, Juiz de Fora, Brazil

**Keywords:** Limnology, Carbon cycle

## Abstract

Freshwater reservoirs, in particular tropical ones, are an important source of methane (CH_4_) to the atmosphere, but current estimates are uncertain. The CH_4_ emitted from reservoirs is microbially produced in their sediments, but at present, the rate of CH_4_ formation in reservoir sediments cannot be predicted from sediment characteristics, limiting our understanding of reservoir CH_4_ emission. Here we show through a long-term incubation experiment that the CH_4_ formation rate in sediments of widely different tropical reservoirs can be predicted from sediment age and total nitrogen concentration. CH_4_ formation occurs predominantly in sediment layers younger than 6–12 years and beyond these layers sediment organic carbon may be considered effectively buried. Hence mitigating reservoir CH_4_ emission via improving nutrient management and thus reducing organic matter supply to sediments is within reach. Our model of sediment CH_4_ formation represents a first step towards constraining reservoir CH_4_ emission from sediment characteristics.

## Introduction

Methane (CH_4_) is a potent greenhouse gas that contributes to climate change with a global warming potential 34 times greater than carbon dioxide (CO_2_) at a 100 year time scale^1^. Methane fluxes from hydroelectric reservoirs, especially from tropical reservoirs, have been debated over the past years^2–4^. Even though hydropower is often thought of as a ‘green’ source of energy, studies suggest that reservoirs are not carbon neutral and in extreme cases might have even higher carbon footprint than fossil fuel energy, particularly those situated in the tropics^5,6^. Tropical reservoirs have been estimated to emit about 3.0 Tg CH_4_-C year^−1 ^^2^ and to contribute 64% of the total reservoir CH_4_ emission^3^. Conversely, latitude was not a strong predictor of reservoir CH_4_ emission in a more recent global analysis^4^. Apparently, the CH_4_ emission from reservoirs is currently not well understood.

At an estimated global organic carbon (OC) burial rate of 0.06 Pg C year^−1^ in reservoir sediments^7^, there is apparently a large supply of organic substrate to the methanogenic microbes that live in anoxic sediments of reservoirs. Important factors that influence rates of CH_4_ formation (net production, i.e. production minus oxidation) in sediment are temperature as well as the organic matter (OM) characteristics (i.e. its reactivity or bioavailability) and OM supply rate. Strong exponential relationships between temperature and CH_4_ formation rates were found in sediments of lakes^8–11^ and rivers^12^, and ecosystem-scale CH_4_ emission strongly depends on temperature^13^. In addition, methanogenesis relies on OM characteristics and supply rate. There is evidence that more CH_4_ is produced from autochthonous OM (derived from aquatic plants and phytoplankton) than from allochthonous OM (derived from land plants and soils)^14^. Also, a high OM supply rate can stimulate high sediment CH_4_ formation and emission, particularly if the OM is biologically reactive^15^. However, allochthonous OM might degrade slower but produce the same amount of CH_4_ over longer period of time^16^, since allochthonous OM contains more support tissues that are processed more slowly^16,17^. When the OM is being decomposed at steady conditions, its CH_4_ formation decreases over time due to rapid initial decay of labile substances^16^. However, at timescales relevant in sediments (years), the effect of decreasing reactivity during OM decomposition on sediment CH_4_ formation is currently not clear. While it has been shown that CH_4_ formation decreases over time^18^, the ageing effect on sediment OM reactivity and CH_4_ formation was not quantified. Therefore, sediment CH_4_ formation can at present not be predicted at environmentally relevant timescales (years), severely limiting our understanding of the globally important function of reservoir sediments as both sinks of OC and sources of atmospheric CH_4_.

Here, we investigate the impact of OM source, characteristics and age on CH_4_ formation rates in sediment of three widely different tropical reservoirs. We performed a long-term incubation experiment (ca 739 days) of sediment varying in age between 1 and 48 years, in order to understand CH_4_ formation in reservoir sediments over the typical lifetime of reservoirs. We selected 3 reservoirs of different productivity and located in different biomes and, therefore, of a range of sediment OM characteristics. We expected a decrease of CH_4_ formation potential with increasing sediment age, and lower CH_4_ formation in the sediment of a reservoir with little autochthonous OM production than in a reservoir with high autochthonous OM production.

## Materials and Methods

### Sampling

Sediment samples were obtained from three Brazilian reservoirs of different trophic status: Chapéu D’ Uvas (CDU), Curuá-Una (CUN) and Funil (FUN). CDU is an oligotrophic reservoir (mean total phosphorus (TP) concentration, 12 µg L^−1^) that was constructed in 1994 for water supply^19^. CUN is a mesotrophic hydroelectric reservoir (mean TP, 19 µg L^−1^) that was constructed in 1977^19^. FUN is a eutrophic hydroelectric reservoir (mean TP 34 µg L^−1^) that was built in 1969^20^. CDU and FUN are located in the Atlantic Forest biome, and CUN is located in the Amazon.

In each reservoir, coring sites (6 in CDU, 4 in FUN and 7 in CUN) were distributed across the entire reservoir (Supplementary Figs 1–3), from the river inflow areas to the dam, to cover gradients in sediment characteristics. Sediment cores (one per site) were taken with a UWITEC gravity corer equipped with a hammer. Tubes of various length (0.6 to 3 m) were used depending on the expected sediment depth. In 14 of 17 sediment cores, we reached the pre-flooding soil. The depth of the interface between pre-flooding soil and reservoir sediment (sediment depth) was visually detected and recorded. If the pre-flooding soil was not reached; sediment depth was estimated from other cores sampled in the same area. Samples from CDU were taken on the 7^th^ of March 2016, from FUN on the 14^th^ of March 2016, and from CUN on the 20^th^–28^th^ of February 2016.

### Sample preparation

Sediment cores were stored in dark at room temperature, i.e. similar to *in-situ* temperatures in these tropical systems, and sliced the day after sampling. Samples were sliced at 4 cm intervals in order to ensure sufficient amount of material for the experiment. The sub-surface (2–6 cm) layer of all sediment cores was used for the experiment to represent rather fresh but already anoxic sediment. We considered 2 cm sediment depth to be anoxic because oxygen penetration depths of 0.3–1.2 cm were reported from a Brazilian reservoir^21^. If possible the 4 cm layer of sediment just above the soil-sediment interface was used and up to 3 additional samples, distributed over the sediment core depth down to the soil-sediment interface, were obtained; for example, the longest core was FUN_48 (204 cm), and the soil-sediment interface was at 192 cm. This core was sampled at 2–6 cm, 32–36 cm, 60–64 cm, 116–120 cm, 172–176 cm. The total number of sediment samples per core varied between one and four, depending on the total length of each sediment core. In total, 17 samples were obtained from 2–6 cm deep sediment, and 25 samples were from deeper sediment layers. After slicing, the headspace in the sample containers (PP jars with LDPE snap lock) was filled with N_2_, and samples were placed in a N_2_-filled glove bag within 1–2 hours after slicing. The glove bag was filled with N_2_ and evacuated using a vacuum pump 2 times to minimize the oxygen exposure of the samples.

Each sample was homogenized in the glove bag, and a sub-sample of 10 mL of homogenized sediment was transferred into pre-weighted 60 mL serum vials. 2.5 or 5 mL (depending on the sediment density) of sterile-filtered (0.2 µm GF filter), N_2_-bubbled water from the respective reservoir was added to the samples. 3 replicates of each sample were prepared. The samples were sealed and flushed with N_2_ after preparation. Vials were weighted before and after filling to know the exact mass of sediment in each vial. As a control treatment, 3 replicates of 10 mL of sterile water (filtered with 0.2 µm GF filter) from each reservoir were prepared in the same vials as sediment sample and sampled in the same way as sediment samples. Dissolved oxygen concentration was checked in those controls at the beginning of incubation with optical sensors (PreSens) and was below detection level (0.1 mg/L detection level).

The rest of the sediment was dried in a custom made oven at about 55 °C, and water content was gravimetrically determined.

### Incubation

Samples were incubated anoxically at 25 °C in the dark throughout the whole experiment time (739 days). The lag phase until the onset of growth of methanogens in similarly handled sediment samples is very short (a few days;^16^) in comparison to the experiment duration, and thus considered negligible. The CH_4_ formation rate was measured in the samples at 7 sampling occasions. Each sampling occasion contained 2 measurements: start and end of incubation, which lasted for about 2 weeks. Exact days of sampling occasions varied for each sample, and were on average day 1–9 for the 1^st^ sampling, day 9–28 the 2^nd^ sampling, day 72–91 for the 3^rd^ sampling, day 185–200 for the 4^th^ sampling, day 275–290 for the 5^th^ sampling, day 423–442 for the 6^th^ sampling and day 719–736 for the 7^th^ sampling (Supplementary Table 3). Before and after each sampling occasion, samples were flushed with N_2_ to prevent any possible inhibition of methanogenesis by CH_4_, CO_2_ or other volatile and potentially inhibitory substances^22,23^. The thermodynamics of metabolic reactions imply a change in free energy in dependence of product concentration^24^, and the resulting negative effect on methanogenic metabolism has been observed in incubation and modelling studies^23,25,26^.

To measure initial gas concentration in the vials (at start of sampling occasions), 8 mL of N_2_ was added to each sample vial, and then pressure in the vial was measured with a needle barometer. Samples were vigorously shaken for 1 min to allow equilibration of CH_4_ between the headspace and the pore-water. Then 7 mL of the headspace gas was extracted in a 10 mL syringe. At the end of each sampling occasion (after about 2 weeks) the procedure was repeated. 8 mL of N_2_ was added to the samples and the gas was extracted after shaking the vial for 1 min. Sample dilution after addition of N_2_ was calculated from pressure difference that was measured before and after the N_2_ addition. Samples were injected the day they were obtained into a GC equipped with a flame ionization detector (FID) (Agilent Technologies, 7890 A GC system). Concentrations of CH_4_ and CO_2_ in the sediment pore water were calculated according to the ideal gas law and Henry’s law, where coefficients for CH_4_ solubility in water at varying temperatures were obtained from Wiesenburg and Guinasso Jr^27^, and coefficients for CO_2_ were obtained from Weiss^28^.

At the end of the experiment, after the last CH_4_ measurement was performed, the sediment was extracted from the serum vials. The sediment of replicates was pooled, mixed and dried in an oven at 60 °C; we decided to pool the replicates because no significant differences were detected in their CH_4_ formation.

The total C (TC) and total N (TN) content in the sediment were measured in each sample at the beginning and end of the incubation with a Costech Elemental Analyzer. We found no significant contribution of inorganic C in our samples.

### Data analysis and calculations

The rates of CH_4_ and CO_2_ formation were obtained by the difference in CH_4_ concentration between two measurements and divided the time interval. The obtained rates represent net CH_4_ formation rates, including both production and any potential consumption of CH_4_ (e.g. by anaerobic CH_4_ oxidation^29,30^) in the sediment.

The age of individual sediment layers was estimated from the multi-year average sedimentation rate, which can be precisely calculated from sediment thickness, and the time elapsed since dam closure, according to the formula:

1 $$sample\,age=\frac{sediment\,layer\,depth}{total\,sediment\,depth}\ast reservoir\,age+incubation\,length$$

where sediment layer depth is the average depth of a slice, total sediment depth is the depth between the sediment surface and the depth of the interface between pre-flooding soil and reservoir sediment. Reservoir age is the time since damming at the sampling day, and incubation length is the time between sediment sampling and the day when CH_4_ formation rate was obtained. Sediment age here represents, then, a measure of time since sediment deposition (and not since OM fixation by photosynthesis or since sediment formation in the aquatic system) assuming constant sedimentation rate. We acknowledge that there probably is year-to-year variability in the sedimentation rate, e.g. due to hydrological extremes, which adds uncertainty to the age estimate of individual sediment layers. However, since there is no reason to assume any systematic change in sedimentation rate with reservoir age, the uncertainty in the estimated age of individual sediment layers is unlikely to suffer from any systematic bias, and is therefore considered to add random error to the age estimates.

Because TC and TN contents were only measured at the start and end of the experiment, we assumed a linear change in TC and TN through the experiment to estimate TC and TN at every CH_4_ measurement occasion. Although it has been shown that the decrease of sediment TC and TN is exponential over timescales of several years-decades^31^, a linear approximation of TC and TN decrease over time is reasonable for relatively short timescales as used in this experiment. Accordingly, when we compared linear TN decrease with exponential TN decrease, modelled using the TN decay rate of 0.16 year^−1^ given by Gälman *et al*.^31^, the difference between TN estimates at any point of time was <0.002% TN, i.e. the potential error of assuming linearity is below analytical precision (data not shown). Since N is preferentially lost over C during sediment organic matter degradation^31^, the effect of interpolation approach on estimated TC will be even smaller than for TN.

In some samples of the deepest sediment layers that had the lowest CH_4_ formation rates, the first 1–2 measurements were excluded because of possible oxygen contamination during sample preparation that delayed the establishment of methanogenic conditions (low microbial activity in old sediment delays the consumption of trace oxygen).

To estimate sediment CH_4_ formation rates over reservoir lifetime we fitted an exponential decay model with 3 OM pools of different reactivity with core as a factor (gnls(CH_4_~a * exp(−b * Age) + c)^32,33^. The significance of the model parameters was assessed with the ANOVA function and the quality of the model was checked by visual examination of the residuals and predicted against measured data. We have also tested other non-linear models (exponential decay model with 2 OM pools, and reactivity continuum model) but they did not converge.

From the exponential decay models we defined a “transition age“ beyond which microbial OM degradation is not quantitatively significant for the standing stock of OC but at a low background level, i.e. the age beyond which sediment OM may be considered “buried” even though microbial activity does not cease entirely. In order to model sediment CH_4_ formation over the typical lifetime of reservoirs (100 years), and in order to estimate the “transition age” to low background CH_4_ formation, we used exponential decay models (described above). We define the transition age as the age at which the slope of the modelled curve is 179°, i.e. almost flat and not distinguishable from 180°, and thus indicative of a constantly low background rate of CH_4_ formation that no longer changes over time (Supplementary Fig. 4). We then integrated CH_4_ formation over the assumed typical lifetime of reservoir of 100 years considering yearly increase in sediment thickness, and calculated the total amount of CH_4_ that would be formed in each sediment core layer over 100 years. We calculated the average and standard deviation of CH_4_ formed for each sampled sediment core separately.

The effect of sample age and TN on the CH_4_ formation rates was modeled with a linear model in R (lm) including the interaction between age and TN (*lm (ln (CH*_4_*) ~ ln(Age)* * *TN*). In the model CH_4_ formation rate per sediment dry weight (µmol g(dw)^−1^ d^−1^) was used because TC and TN are dependent on each other. The CH_4_ formation rates and sample age were ln transformed. The model assumptions were checked through model residual plots.

Correlation between transition age and C:N ratio was checked with a Pearson correlation. The difference between fresh and old sediment CH_4_ formation was checked with repeated measures ANOVA. Both model assumptions were checked through model residual plots. We used R (R 3.4.3^34^) for data analysis, modelling and plotting.

## Results

### Sediment characteristics

The sediment samples used in our study differed in sediment characteristics (Table 1). The Amazonian CUN reservoir had the highest mean TC content (6.6%) as well as the highest mean C:N ratio (15.2), while the eutrophic FUN reservoir showed the lowest values (2.5% TC and C:N 10.6, Table 1). TN content was in general similar among the reservoirs, with slightly higher values in CUN (Table 1).

**Table 1:** Pre-incubation sediment water content, total carbon content, total nitrogen, C:N ratio and total number of sediment slices used for the experiment (n) in the 3 studied reservoirs (mean (min–max)).

Reservoir	% water	% TC	% TN	C:N ratio	n
CDU	66 (48–81)	3.5 (0.9–10.2)	0.3 (0.1–0.8)	13.7 (11.4–16.4)	12
FUN	66 (53–81)	2.5 (1.8–4)	0.3 (0.2–0.4)	10.6 (9.1–13.0)	14
CUN	73 (42–87)	6.6 (2.3–12.3)	0.5 (0.1–0.8)	15.2 (11.2–23.6)	14

### CH_4_ formation rates measured during the incubation experiment

**Figure 1:**
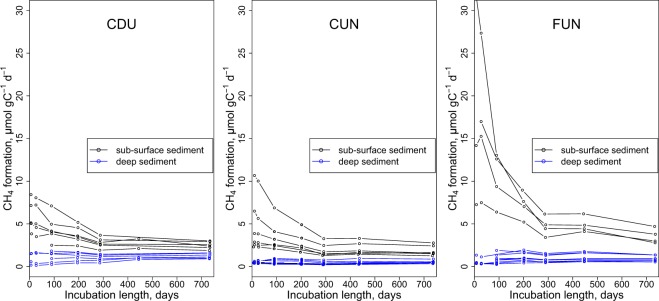
CH_4_ formation rates over the time of the incubation experiment in the three reservoirs. Black lines are sub-surface sediment (2–6 cm depth) and blue lines are deeper sediment. Circles are an average of three replicates of CH_4_ formation rates with a mean stdev of 8.6% of the mean.

CH_4_ formation per sediment dry weight (µmol g(dw)^−1^ d^−1^) significantly correlated with the amount of TC in the sediment (R^2^ = 0.56, p < 2.2^−16^, data not shown) therefore CH_4_ formation rates were normalized for TC (µmol gC^−1^ d^−1^). There was a significant difference between CH_4_ production in sub-surface (2–6 cm) and deeper sediment layers (p < 2^−16^, Fig. 1).

CH_4_ formation rates decreased over time of the 739 d incubation experiment in sub-surface sediment layers (2–6 cm), but remained rather stable in deeper sediment layers (Fig. 1). CO_2_ formation rates also decreased over time for sub-surface sediment layers and also for deeper sediment for FUN (Supplementary Fig. 5). The highest CH_4_ formation rate was measured in a FUN sub-surface sediment sample (core FUN_46 at the first measurement occasion at day 7: 30.4 ± 2. µmol gC^−1^ d^−1^ (mean ± sd)). At the last measurement at day 736, the same sample had CH_4_ formation rate of 2.4 ± 0.1 µmol gC^−1^ d^−1^. On average FUN experienced highest CH_4_ formation rates in sub-surface sediment over the whole incubation period (8.8 ± 7.0 µmol gC^−1^ d^−1^, (mean ± sd)), while in CUN and CDU mean CH_4_ formation rates in sub-surface layers were lower over the whole incubation period (2.7 ± 2.3 µmol gC^−1^ d^−1^ and 3.9 ± 1.8 µmol gC^−1^ d^−1^ respectively). CH_4_ formation rates decreased most rapidly in FUN sub-surface sediment over the time of the incubation. The final CH_4_ formation rates (day 739) in sub-surface sediment in FUN were 24 ± 12% of the initial rates, while in CUN and CDU the CH_4_ formation rates decreased less and were 50 ± 16% of the initial rates in CUN and 50 ± 15% in CDU.

CH_4_ formation rates of deepest sediment were significantly different between reservoirs (p < 0.001, Kruskal – Wallis test). CDU had higher mean CH_4_ formation rates over the whole incubation period in bottom layers (1.07 ± 0.49 µmol gC^−1^ d^−1^) than FUN (0.44 ± 0.14 µmol gC^−1^ d^−1^) and CUN (0.44 ± 0.16 µmol gC^−1^ d^−1^).

### CH_4_ formation rates over sediment age

**Figure 2:**
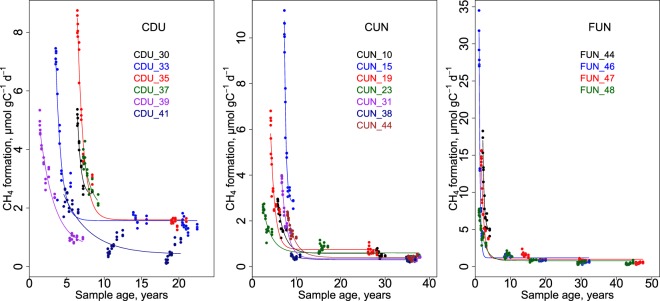
CH_4_ formation rates in sediment over age in the 3 reservoirs. Lines and points of the same colour are samples taken from the same sediment core, and correspond to the colour of the core name on the legend. Lines are exponential decay models of the cores (model statistics are found in Supplementary Table 1). Note the differences in scales.

CH_4_ formation rates decreased exponentially with sediment age (Fig. 2). In none of the samples, the CH_4_ formation rate was zero.

The decrease of CH_4_ formation rate over time was most pronounced in FUN (Fig. 2). The highest CH_4_ formation in FUN was measured in 1 year old sediment (30.4 ± 2.7 µmol gC^−1^ d^−1^) and the lowest in 42.9 years old sediment (0.2 ± 0.005 µmol gC^−1^ d^−1^). We could also observe clear exponential decrease in sediment CH_4_ formation in CUN, where the estimated age of the samples varied from 2 to 38 years. In CUN, the highest CH_4_ formation of 11.4 ± 0.6 µmol gC^−1^ d^−1^ was measured at 7 years old sediment and the lowest CH_4_ formation rate was measured in 36 years old sediment (0.2 ± 0.01 µmol gC^−1^ d^−1^). CDU is the youngest of the studied reservoirs. Estimated sediment age in CDU varied from 1 to 22 years. The highest CH_4_ formation in CDU was measured at 6 years old sediment (8.0 ± 0.2 µmol gC^−1^ d^−1^) and the lowest was 0.1 ± 0.03 2 µmol gC^−1^ d^−1^ at 18 years old sediment.

Models for CH_4_ formation over time achieved best fit on deeper sediment cores where a wide age gradient was covered by many samples. In cases where the age gradient was very short, model fits were sometimes poor. Model statistics and coefficients can be found in Supplementary Table 1.

### CH_4_ formation rates as a function of TN and sediment age

**Figure 3:**
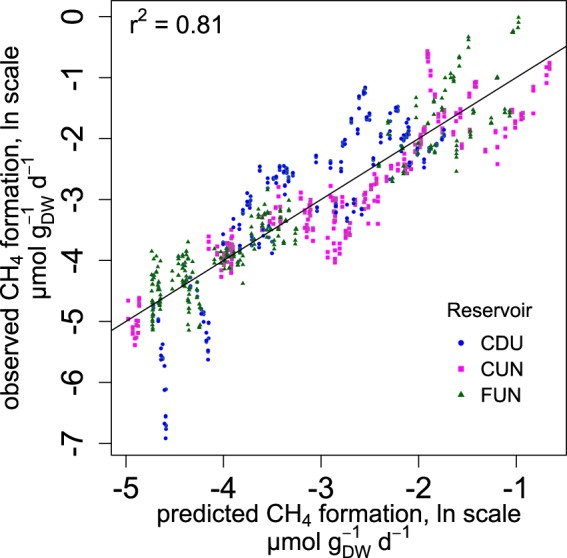
CH_4_ formation (in ln scale) in all sediment samples as a function of TN and ln(Age). The line is the 1:1 line.

Merging all 764 measured CH_4_ formation rates in these three widely different reservoir sediments (Table 1) into one dataset, we found that CH_4_ formation rates could be predicted from the sediment TN (%) and the age of the sediment (R^2^ = 0.81, p < 2^−16^; Fig. 3; see Supplementary Table 2 for model statistics):

2 $$\mathrm{ln}({{\rm{CH}}}_{{\rm{4}}}\,{\rm{formation}})=-\,0.59\ast \mathrm{ln}({\rm{Age}})+6.46\ast TN-0.99\ast \mathrm{ln}({\rm{Age}})\ast {\rm{TN}}-{\rm{3.12}}$$

where CH_4_ formation is expressed in µmol g(dw)^−1^ d^−1^, TN is expressed in mass %, and age is expressed in years.

The back-transformation of ln-transformed predictions renders residuals that are heavily skewed, leading to underestimation of predicted mean CH_4_ formation rate^35^. Therefore we corrected the predicted CH_4_ formation rate following^36^:

3 $${{\rm{CH}}}_{4}\,{{\rm{formation}}}_{{\rm{corr}}}=\exp (\mathrm{ln}\,{{\rm{CH}}}_{4}\,{\rm{formation}}+0.5\ast {{\rm{s}}}^{2})$$

4 $${{{\rm{s}}}^{2}}_{{\rm{corr}}}={({{\rm{CH}}}_{4{\rm{corr}}}{\rm{formation}})}^{2}\ast \exp ({{\rm{s}}}^{2})-1)$$

where CH_4_ formation is the ln transformed predicted CH_4_ formation rate and s^2^ = 0.28 is a residual variance of the model. CH_4_ formation _corr_ is the corrected CH_4_ formation rate and s^2^_corr_ is the corrected variance.

## Discussion

We report here the first model of CH_4_ formation in freshwater sediment that is applicable at relevant timescales (Fig. 3). Accordingly, CH_4_ formation rates stretching over more than 2 orders of magnitude could be predicted for sediments that cover a wide gradient in sediment characteristics (Table 1) and span over decades in age. Sediment age (i.e. time since sediment deposition) was an important predictor of CH_4_ formation (Supplementary Table 2), which can be seen in the exponential decrease of CH_4_ formation as the sediment got older, both in sub-surface sediment samples during our 739 days of experiment (Fig. 1), and when comparing sediment layers along a decadal age gradient (Fig. 2). We note that age was important in spite of the random noise inflicted by the unknown year-to year variability in sediment accumulation on the age estimate of individual sediment layers. The sediment TN concentration was the other important predictor of the CH_4_ formation model, which explained 81% of the variability in CH_4_ formation rates (Fig. 3). Even though a significant relationship between TN and CH_4_ formation in freshwater sediment was previously reported^18^, no relation to sediment age has been made beyond the timescales of incubation experiments (e.g.^18^). Contrarily, our analysis revealed that not only the TN concentration was important for CH_4_ formation, but also the change of TN over sediment age (i.e. the interaction term age * TN; Eq. 2). We suggest that the importance of TN may be related to the amount, source and diagenetic state of the sediment OM. First, we found that in our dataset, the sediment TN concentration was also positively related to the TC concentration (TN = 0.067 * TC + 0.077, p < 2.2^−16^, R^2^ = 0.90, not shown). Second, the TN concentration in the sediment is representative of the OM source^37^: autochthonous OM is comparatively rich in protein and poor in cellulose (i.e. high in TN), while allochthonous OM is protein-poor and rich in lignocellulose (i.e. low in TN). It was previously shown that N-rich autochthonous OM is more biodegradable^38^ and produces more CH_4_ than the degradation of allochthonous OM^14^ (even if very fresh allochthonous OM can also give rise to substantial CH_4_ production^16^), explaining the relationship between sediment TN for CH_4_ production (Eq. 2;^18^). Third, N is preferentially consumed during microbial degradation of sediment OM (e.g.^31^), meaning that over time, the sediment OM becomes poorer in TN and less reactive, in accordance with our finding that the interactive term (age * TN) was significant in the model. In fact, also sediment age is a proxy of OM reactivity, given the steep exponential decrease of the sediment OC decay with increasing sediment age^39^. The model presented here for the first time quantifies the influence of OM input (amount and source) and its diagenetic change, approximated by age, TN concentration and their interaction, on CH_4_ formation rates from sediment OM over decal timescales. In addition, the model is derived from tropical reservoirs of different trophic status and biomes, and in spite of the considerable variability of CH_4_ formation between reservoirs, all predicted CH_4_ formation rates for all cores from all reservoirs converge on one line (Fig. 3). For this reason, it seems likely that this relationship is valid in tropical reservoirs in general, and that CH_4_ formation can be predicted from TN and age, factors that can be relatively easily measured or approximated. Importantly, the rates presented here were derived at a standardized temperature of 25 °C. In order to derive *in-situ* CH_4_ rates, our model needs to be used in conjunction with the well-documented temperature dependence of CH_4_ production^40^. Also, before applying it to other systems, or beyond the data domain from which it was constructed, the model first needs to be verified.

CH_4_ formation decreased over age in all sediment cores, however, the CH_4_ formation never reached zero, not even at over 40 years of age. Despite that CH_4_ formation slows down rapidly after sediment is deposited, CH_4_ continues to be produced in older sediment layers. For this reason, it is strictly speaking not warranted to speak of OC burial in reservoir sediment, simply because it is in many cases relatively young and thus still being degraded. However, we may determine the age at which CH_4_ formation asymptotically reaches a stable and low ‘background’ rate. Sediment younger than that may still be considered to degrade significantly, while sediment older than this threshold may be considered largely stabilized, i.e characterized by low and stable CH_4_ formation (see Methods). This age may therefore serve as an operationally defined age beyond which sediment OC may be considered “buried”, i.e. not degraded to an extent that is quantitatively significant for the standing stock of OC. The age of transition to low background CH_4_ formation calculated by this approach was about 11 years in CDU and 12 years in CUN and 6 years in FUN (Table 2), which corresponds to 13, 9 and 23 cm of sediment depth in CDU, CUN and FUN respectively. Assuming a 100 year lifetime for these reservoirs, around 38% (Table 2) of the total time-integrated sediment CH_4_ formation would take place in sediment layers that are beyond the transition age threshold and that have reached a low and stable background CH_4_ formation. In other words, sediment layers that we often consider biologically inactive might significantly contribute to the overall sediment CH_4_ production.

**Table 2:** Age of transition to low background CH_4_ formation, defined as the age at which the slope of the exponential decay curve reaches 179° (see Methods), and the corresponding sediment depth.

Reservoir	Transition age (years)	Transition depth (cm)	% of CH_4_ formed in sediment older than transition age
CDU	11.4 ± 3.2	13 ± 11	38 ± 25
CUN	11.9 ± 2.1	9 ± 4	39 ± 26
FUN	6.4 ± 2.5	23 ± 11	35 ± 24

The contribution of sediment layers beyond the transition age to total CH_4_ formation over the lifetime of the reservoirs assumes 100 year lifetime.

Despite a significant contribution of older sediment layers to CH_4_ formation (with an average measured CH_4_ formation in sediment older than the transition age for 179 degree criteria 1.2 ± 0.6 µmol gC^−1^ d^−1^ in CDU, 0.5 ± 0.2 µmol gC^−1^ d^−1^ in CUN and 0.8 ± 0.1 µmol gC^−1^ d^−1^ in FUN), the amount of C that is degraded through the CH_4_ formation is quantitatively negligible compared to typical carbon burial rates. According to our measured CH_4_ formation rates in sediment older than the transition age, on average 0.3 ± 0.2% of the C contained in a sediment layer is transformed into CH_4_ in a year.

**Figure 4:**
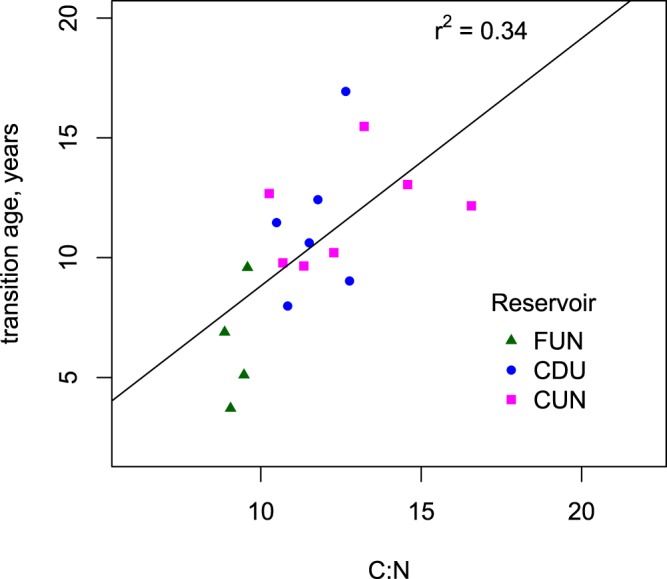
The transition age in all 3 reservoirs vs C:N ratio of surface sediment of the same sediment core as the transition age was determined for (surface sediment CN ratio used as a proxy of reactivity of C that is deposited onto the sediment surface).

We used the C:N ratio of the surface sediment to evaluate if the source of the sediment deposited onto the sediment surface has an effect on the transition age (Fig. 4). We found a significant increase in the transition age with increase in C:N ratio (p = 0.012, R2 = 0.34, n = 17). This indicates that autochthonous OM (low C:N) is relatively easily decomposed and readily transformed into CH_4_^16^, but once the labile substances are decomposed the degradation rate declines steeply (Fig. 2). Allochthonous OM (high C:N) on the other side breaks down more slowly than autochthonous OM, and the slow anaerobic decay of refractory fraction of allochthonous OM can provide a continuous source of CH_4_ in reservoir sediment over the reservoir lifetime.

### Implications

Our study shows that the CH_4_ formation rate of reservoir sediments is predictable although the fluxes may further vary with sediment temperature and dissolved oxygen concentration^40^. As CH_4_ emission from reservoirs is very difficult to measure representatively due to the strong spatial and temporal variability of CH_4_ emission via diffusion^19^, and particularly via ebullition^41,42^, the sediment CH_4_ formation model (Eq. 2, Fig. 3) represents the first step towards constraining reservoir CH_4_ emission from sediment characteristics.

Further, our study indicates that the high CH_4_ emission from eutrophic reservoirs^4^ may stem primarily from CH_4_ formation in young sediment layers (e.g. FUN, <6 years old; Table 2). Hence, reservoir management strategies that decrease nutrient inputs to limit autochthonous primary production could within less than a decade lead to a reduction in reservoir CH_4_ emission to the atmosphere.

Lastly, the current hydropower boom in tropical areas^43^ carries a risk of creating new reservoirs that are large CH_4_ emitters: the high potential for soil erosion in the tropics^44,45^ implies that new tropical reservoirs may have high sedimentation rates and thus high supply of young and reactive OM that stimulates CH_4_ formation in the reservoir sediment (Fig. 2). This effect is aggravated in case nutrient management strategies are lacking and excess nutrient loads stimulate productivity, since the transformation of autochthonous OC to CH_4_ in reservoir sediment results in an anthropogenic enhancement of atmospheric radiative forcing^46^.

## Supplementary information

Supplementary info

## Data Availability

The data are be publicly available on the DiVA repository http://urn.kb.se/resolve?urn=urn:nbn:se:uu:diva-387547).
