# Supplementary material for: Methane formation in tropical reservoirs predicted from sediment age and nitrogen
Source: Sci Rep. 2019 Jul 29;9:11017. doi: 10.1038/s41598-019-47346-7 (PMC6662704; doi:10.1038/s41598-019-47346-7)
Supplement: Supplementary file 1 — Supplementary info [file 41598_2019_47346_MOESM1_ESM.pdf]

Supplementary information for

Methane formation in tropical reservoirs predicted from sediment age and nitrogen

Anastasija Isidorova[1], Charlotte Grasset[1], Raquel Mendonça[1,2], Sebastian Sobek[1]

[1] Limnology, Department of Ecology and Genetics, Uppsala University, Uppsala, Sweden
[2] Laboratory of Aquatic Ecology, Department of Biology, Federal University of Juiz de Fora, Juiz de Fora, Brazil

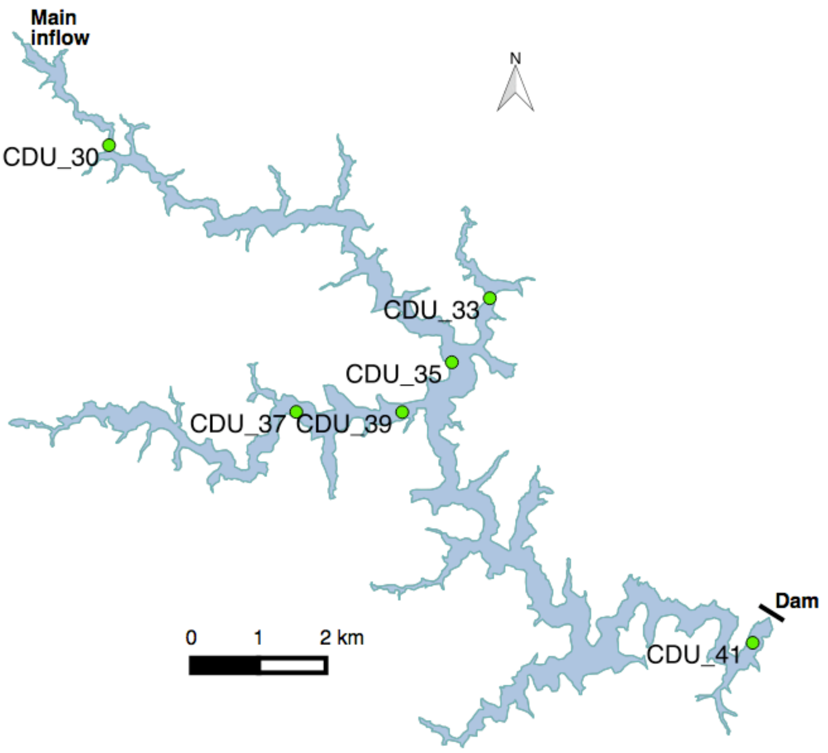

Figure S1. *Chapeu D'Uvas (CDU; 21°35'06.9"S 43°31'47.8"W) sampling sites.*

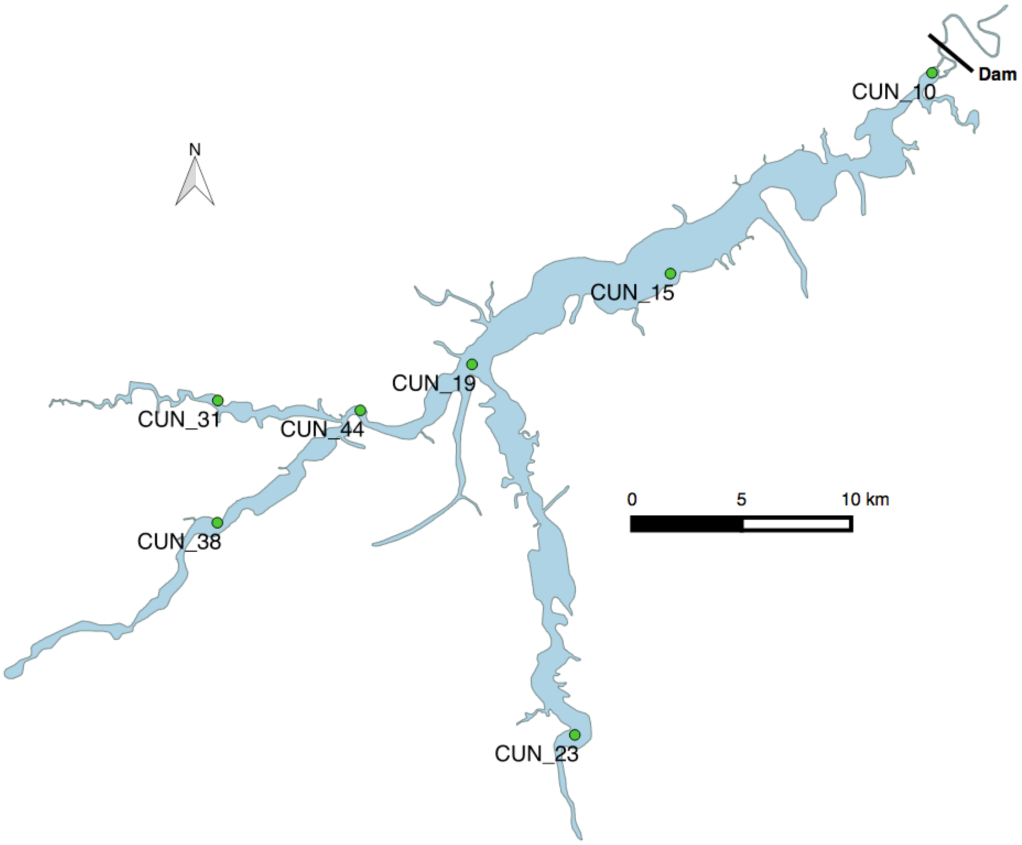

Figure S2. *Curua Una (CUN; 2°49'03.5"S 54°18'11.3"W)* sampling sites

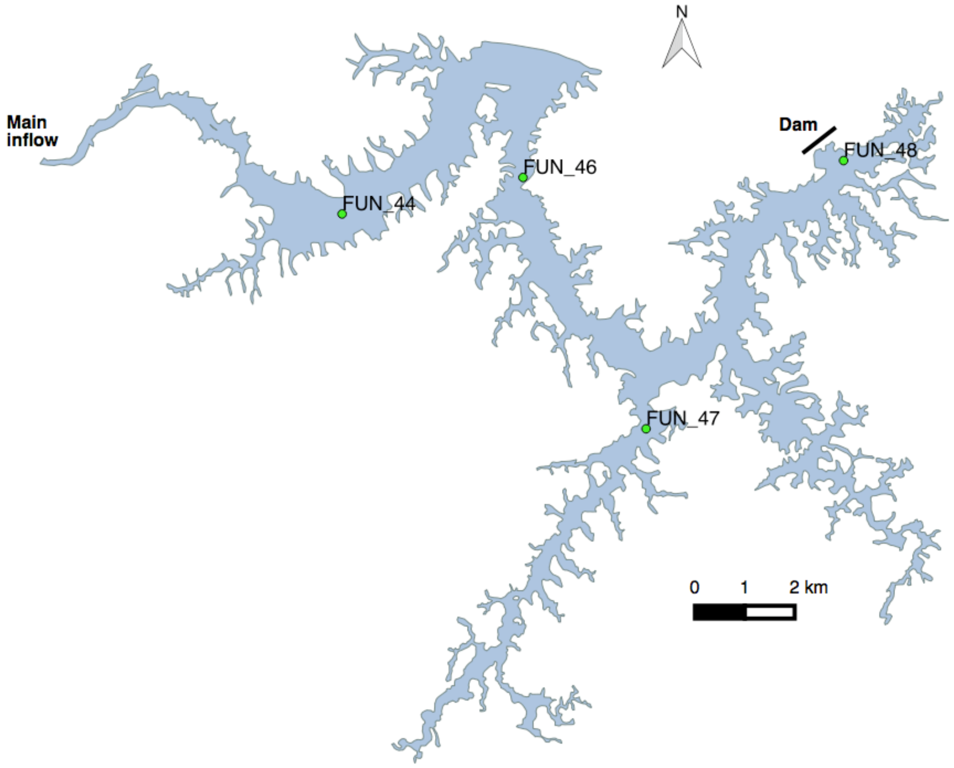

Figure S3. *Funil (FUN; 22°31'44.0"S 44°33'46.7"W) sampling sites.*

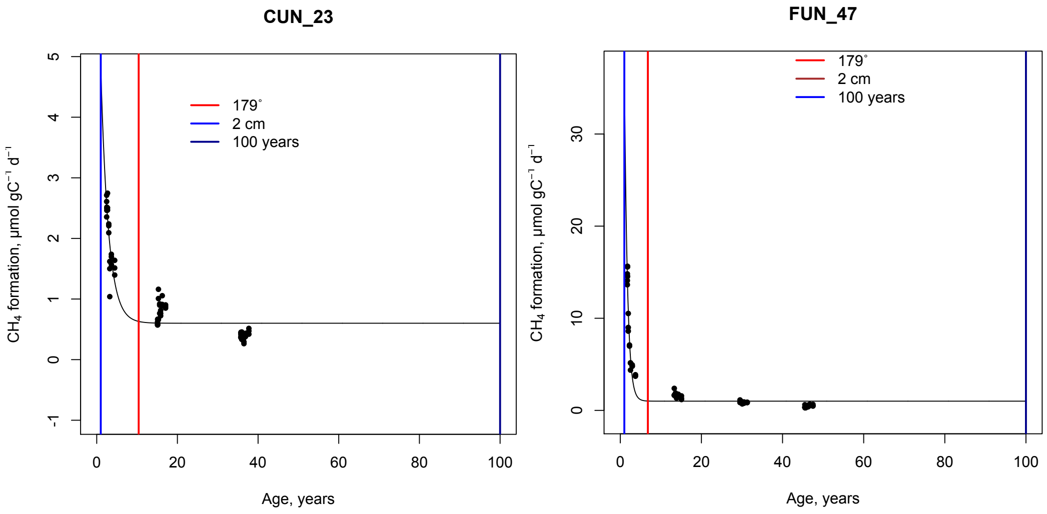

Figure S4. *Example of how sediment age differed at 179° criteria applied for 2 sediment cores*

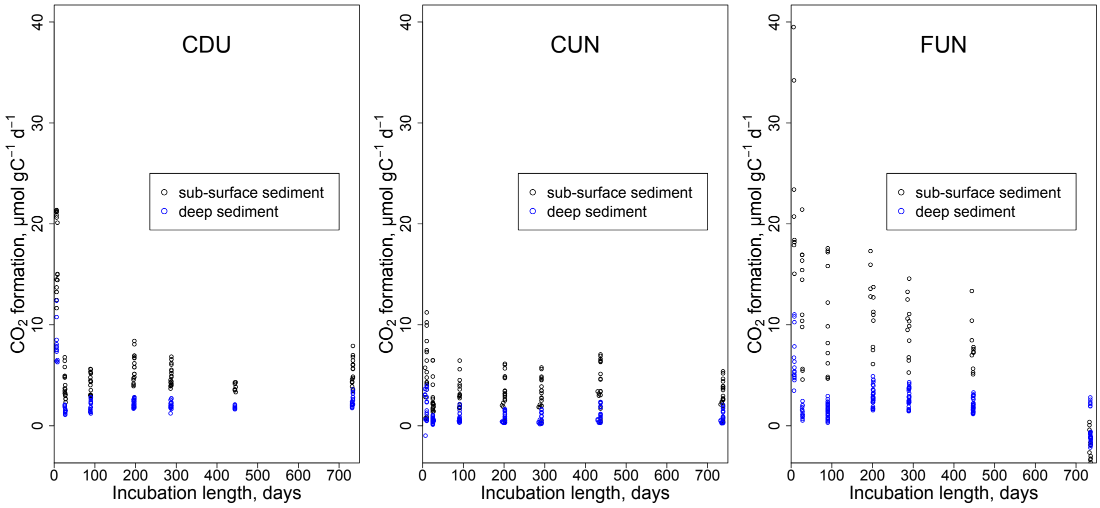

Figure S5. *CO_2 formation rates over the time of the incubation experiment in the three reservoirs. Black points are sub-surface sediment (2-6 cm depth) and blue points are deeper sediment.*

Table S1. Parameters of the exponential decay model of the decomposition of OC into CH_4

Core	a	b	c
CDU_30	3.3E+06 ± 1.7E+07 ^{ns}	2.2 ± 0.8 **	2.4 ± 0.4 ***
CDU_33	6.7E+02 ± 4.2E+02 ^{ns}	1.4 ± 0.2 ***	1.6 ± 0.1 ***
CDU_35	1.1E+04 ± 8.8E+03 ^{ns}	1.1 ± 0.1 ***	1.6 ± 0.1 ***
CDU_37	2.9E+02 ± 1.7E+03 ^{ns}	0.7 ± 0.9 ^{ns}	1.7 ± 1.5 ^{ns}
CDU_39	8.2E+00 ± 1.3E+00 ***	0.5 ± 0.1 ***	0.6 ± 0.3 ^{ns}
CDU_41	7.5E+00 ± 3.3E+00 *	0.3 ± 0.1 **	0.4 ± 0.2 ***
CUN_10	8.3E+01 ± 9.7E+01 ^{ns}	0.6 ± 0.2 **	0.6 ± 0.1 ***
CUN_15	1.9E+10 ± 5.0E+10 ^{ns}	3.0 ± 0.4 ***	2.8 ± 0.3 ***
CUN_19	2.9E+02 ± 1.6E+02 ^{ns}	1.0 ± 0.1 ***	0.7 ± 0.1 ***
CUN_23	6.8E+00 ± 3.5E+00 ^{ns}	0.5 ± 0.2 **	0.6 ± 0.1 ***
CUN_31	6.9E+02 ± 7.0E+02 ^{ns}	0.8 ± 0.1 ***	0.4 ± 0.1 **
CUN_38	1.5E+00 ± 2.4E+01 ^{ns}	0.3 ± 1.7 ^{ns}	0.3 ± 0.1 *
CUN_44	1.0E+02 ± 1.7E+02 ^{ns}	0.5 ± 0.2 *	0.4 ± 0.1 **
FUN_44	3.2E+03 ± 1.5E+03 *	2.6 ± 0.2 ***	4.9 ± 0.3 ***
FUN_46	1.0E+03 ± 9.3E+01 ***	3.2 ± 0.1 ***	1.2 ± 0.1 ***
FUN_47	1.5E+02 ± 1.8E+01 ***	1.4 ± 0.1 ***	1.0 ± 0.1 ***
FUN_48	1.3E+01 ± 1.2E+00 ***	0.6 ± 0.1 ***	0.8 ± 0.1 ***

Residual standard error: 0.57
Degrees of freedom: 764 total; 713 residual
a, b and c are the parameters given by the exponential decay model
Significance levels of the parameters are * p<0.05, ** p<0.01, *** p<0.001, ns- not significant
Model (in R): gnls(CH4~a*exp(-b*Age)+c, start=list(a=coefficients$a, b=coefficients$b, c=coefficients$c), param=list(a~Core-1,b~Core-1,c~Core-1))

Table S2 CH_4 production rates as a function of TN and sediment age model statistics

	Estimate	Std. Error	t value	Pr(>|t|)
(Intercept)	-3.11572	0.14224	-21.905	< 2e-16
ln(Age)	-0.58876	0.05025	-11.717	< 2e-16
TN	6.46119	0.39081	16.533	< 2e-16
ln(Age):TN	-0.98696	0.13294	-7.424	3.04E-13

RMSE (residual standard error)	0.5454
df	760
Adjusted R2	0.8078
Multiple R2	0.8086
p-value	< 2e-16

Table S3. Range of start and end days of sampling occasions.

Sampling occasion	Start day range	End day range
1	1 – 7	7 – 11
2	7 – 11	26 – 29
3	70 – 77	90 – 92
4	180 – 187	196 – 203
5	271 – 278	286 – 293
6	419 – 426	431 – 449
7	714 - 722	733 - 739
